# Supplementary material for: What integrated care means from an older person’s perspective? A scoping review protocol
Source: BMJ Open. 2018 Mar 8;8(3):e019256. doi: 10.1136/bmjopen-2017-019256 (PMC5855209; doi:10.1136/bmjopen-2017-019256)
Supplement: Supplementary data [file bmjopen-2017-019256supp001.pdf]

Appendix 1- PRISMA-P Checklist

Section and topic	Page number
**Administrative Information**	
Title:	
Identification	1
Update	N/A
Registration	N/A
Authors:	
Contact	1
Contributions	9
Amendments	N/A
Support:	
Sources	9
Sponsor	9
Role of sponsor or funder	9
**Introduction**	
Rationale	4-5
Objectives	5
**Methods**	
Eligibility criteria	6-7
Information sources	6-7
Search Strategy	7-8
Study records:	
Data management	7-8
Selection process	7
Data collection process	8
Data items	
Outcomes and prioritization	8-9
Risk of bias in individual studies	
Data synthesis	8
Meta-bias(es)	N/A
Confidence in cumulative evidence	N/A
