# Supplementary material for: What integrated care means from an older person’s perspective? A scoping review protocol
Source: BMJ Open. 2018 Mar 8;8(3):e019256. doi: 10.1136/bmjopen-2017-019256 (PMC5855209; doi:10.1136/bmjopen-2017-019256)
Supplement: Author's manuscript [file bmjopen-2017-019256.draft_revisions.pdf]

BMJ Open

BMJ Open is committed to open peer review. As part of this commitment we make the peer review history of every article we publish publicly available.

When an article is published we post the peer reviewers' comments and the authors' responses online. We also post the versions of the paper that were used during peer review. These are the versions that the peer review comments apply to.

The versions of the paper that follow are the versions that were submitted during the peer review process. They are not the versions of record or the final published versions. They should not be cited or distributed as the published version of this manuscript.

BMJ Open is an open access journal and the full, final, typeset and author-corrected version of record of the manuscript is available on our site with no access controls, subscription charges or pay-per-view fees (http://bmjopen.bmj.com).

If you have any questions on BMJ Open's open peer review process please email info.bmjopen@bmj.com

What integrated care means from an older person's perspective? A scoping review protocol

Journal:	*BMJ Open*
Manuscript ID	bmjopen-2017-019256
Article Type:	Protocol
Date Submitted by the Author:	23-Aug-2017
Complete List of Authors:	Mittinty, Manasi; The University of Adelaide, Adelaide Nursing School<br>Marshall, Amy; The University of Adelaide, Adelaide Nursing School<br>Harvey, Gillian; University of Adelaide
**Primary Subject Heading**:	Geriatric medicine
Secondary Subject Heading:	Evidence based practice, Geriatric medicine, Health services research, Patient-centred medicine
Keywords:	integrated care, ageing population, older people, person-centred

SCHOLARONE™
Manuscripts

For peer review only

What integrated care means from an older person's perspective? A scoping review protocol

Manasi Murthy Mittinty[1], Amy Marshall[1] and Gillian Harvey[1].

[1] Adelaide Nursing School, The University of Adelaide, Adelaide, South Australia, 5000

Corresponding author: Manasi Murthy Mittinty
Email: manasi.mittinty@adelaide.edu.au
Address: Level 4, AHMS building, 57 North Terrace, Adelaide, South Australia, 5005

For peer review only

Abstract

Introduction: According to the 2013 WHO Global Forum on Innovation for Ageing Populations many disabilities and morbidities associated with ageing are often preventable by accessing preventive care. One of the identified ways of preventing multi-morbidities in the older population is the provision of "integrated care". Although integrated care means different things to different people, it typically symbolises continuity in care, thus preventing older patients from falling through gaps in the health and care system. However, very little is known about patient expectations and experiences of integrated care, which is vital for developing and implementing better models of care. The proposed scoping review aims to map literature on older patients' views, expectations, experiences and perspectives of integrated care.

Methods and analysis: Multiple electronic databases including PubMed, Web of Science, Embase, PsychInfo, Google Scholar, Cochrane Library, CINHAL, and ProQuest Dissertations will be searched for appropriate articles. Reference lists of selected articles will also be manually searched for similar articles. Two experienced researchers will conduct an initial search of the literature to identify relevant articles. Abstracts of the identified articles will be reviewed collectively by two researchers to identify potential further studies. Full texts of the potential studies will be sourced and screened for the inclusion criteria. Appropriate qualitative and quantitative methods will be used to extract data from each included study.

Ethics& Dissemination: The scoping review will synthesize findings from studies reporting on patients' views and expectations of integrated care. This review expects to find information relating to facilitators and barriers of integrated care from an older person's perspective. The findings from this review will be applied when working with stakeholders representing older people, health care, aged care and community providers, researchers and policy makers to develop and test a more locally tailored and person-centred approach to integrated care.

Keywords: integrated care; ageing population; older people; person-centred

For peer review only

Strengths and limitations of the study

- This study will be the first scoping review to provide comprehensive understanding of what older people consider to be integrated care; what does it mean to them and what do they expect?
- This study will search all sources of literature covering peer-reviewed articles, unpublished reports, conference proceedings and bibliographies
- Stakeholders involved in provision of or affected by integrated care like older patients, health care providers, government organisations, carers and nurses will be engaged throughout the review process.
- Scoping reviews are generally not considered to provide generalizable findings because of the lack of synthesis of results; conducting a thematic analysis of the available literature will go some way to providing further insight into the findings than descriptive data alone.
- As this review will incorporate studies from different health care settings, it may lack specificity.

For peer review only

Background:

In the last 40 years, life expectancy has increased significantly on a global level[1 2] with a resultant increase in the proportion of older people in the population. This shift in demographics has both benefits and challenges. Older people are a great social and cultural resource; they are also lower financial contributors and higher health care system users. As older people often suffer from multi-morbidity, they typically receive treatments from more than one health professional, consume multiple medications and have multiple organisations and service providers involved in their care.[3] As a result, older people tend to have more preventable acute hospitalisations,[4-6] which puts significant strain on the patients, their families, carers, and health care systems resulting in suboptimal health outcomes and reduced quality of life for patients and increased health care costs.[7] These acute hospitalizations are frequently linked with a lack of integrated care.[8] Integrated care is considered a logical way to organize health care to reduce demands on the local health services while generating a broad range of services for patients.

Providing integrated, patient-centred care promises to improve patient experience while minimizing the unnecessary use of healthcare resources.[9] However, it represents a major challenge facing health service providers and policy makers.[10] Despite numerous attempts at integration, uncertainty remains about which approaches are most effective [9] or how to achieve an appropriate form of integration at a local level.

Though there is a growing body of scientific evidence relating to integrated care and its implications, very few studies [11 12] have summarized patients' perspectives and expectations of integrated care. There is a lack of comprehensive understanding of what older people themselves consider to be integrated care; what does it mean to them and what do they expect? Successful management of chronic conditions heavily depends upon matching clinical evidence with patient expectation and experience.[13] There suggests an important need for understanding patients' perspectives and views of integrated care. The proposed scoping review aims to map literature on patients' views, expectations, experiences and perspectives of integrated care. This paper describes the protocol that will be followed for conducting the scoping review.

Study rationale:

Globally, a large number of heterogeneous, disease-specific and setting-specific models of integrated care are being implemented. However it remains unclear the extent to which these

For peer review only

models have been validated, in terms of meeting a patient-centred view of integrated care. The necessity to incorporate patients' views when building these models has been identified by several studies.[14-16] Currently, there is no information on what factors patients' perceive to support or hinder their care. To our knowledge, there is only 1 study published on synthesis of patient experiences and expectations of integrated care.[17] In the absence of a clear person-centred definition of integrated care, the scoping review seeks to facilitate the development of such a definition. It will also provide insight into this poorly understood area of research, and help to map the characteristics of the primary research that currently exists, as well as identifying knowledge gaps.

The objective of the review is to systematically examine the range, and scope of literature; and to determine enablers and barriers of integrated care from the patients' perspectives. This work will constitute the first step in a multi-stage Knowledge Translation study, to be conducted in South Australia (SA), which aims to design, implement and evaluate an evidence-based, locally relevant and person-centred approach to integrated care for frail older people. The scoping review will allow to build an in-depth understanding of person centred integrated care and the contextualised barriers and enablers from the patients' perspectives. This information will inform the co-design, implementation and evaluation of a person-centred integrated model of care for older people who are frequent users of potentially preventable acute care. In turn this information will allow policy makers and healthcare providers to modify their services to patient demands and needs.

Research Methods:

Literature search

A pragmatic publication date cut-off point of June 2008 was adopted to focus on studies that were conducted after the publication of a working definition of integrated care by WHO.[14] The scoping review will use the six steps described by Arskey and O'Malley [18] for conducting a scoping review. These steps will be used as a guide to identify, select and review the literature.

Step1: Identifying the research question

Integrated care is defined as, "*The management and delivery of health services so that clients receive a continuum of preventive and curative services, according to their needs over time and across different levels of the health system",* WHO, 2008. [19] This definition suggests that

For peer review only

a wide array of health care activities – including, but not limited to, frequency of consultation, quality of consultation, location of medical services, interaction with healthcare providers, satisfaction with the care provided to follow up plans - form integrated care. Therefore, an iterative approach will be adopted for refining the research questions. This approach will allow us to familiarize with the current literature on the topic; to synthesize knowledge and information from relevant studies while helping to identify the knowledge gaps. Broad research question that we will start with is ….

"What are the findings of the research on older patient views, perspectives, expectations and experiences of integrated care?"

Step 2: Identifying the relevant studies

The literature search will be performed from August 2017– October 2017, using PubMed, Web of Science, Embase, Google Scholar, Cochrane Library, CINHAL, and ProQuest Dissertations and Theses Global. Terms specific to each individual database will be used for searching articles. Selection criteria defined in Table 1 will be followed for inclusion or exclusion of articles in the scoping review.

Table 1: Selection criteria to be used for identifying studies.

Study selection criteria	• Articles in English language published between June 1, 2008-October 31st 2017;<br>• Studies conducted or reporting only on human subjects.<br>• Studies reporting on empirical, interpretive and critical research using any type of study methodology or study designs (case control study, observational study, surveys, research reports and case reports)<br>• Studies reporting on any types of health care setting like primary care, hospitals, allied health practices, or emergency hospitals.
Participant selection criteria:	• Studies conducted or reporting only on participants aged 60 years and above.<br>• There will be no limitation on upper age and gender of the participants.<br>• There will be no limitations on geographical location of the

For peer review only

	study participants.
Specific exclusion criteria:	• Studies reporting on non-human subjects.<br>• Studies not reporting on individuals aged 60 years and above.<br>• Studies reported in another language than English.

Step 3: Study selection

Two experienced researchers will independently conduct an initial search of literature to identify relevant articles. A priori set of search terms will be formulated prior to conducting the initial search. A full list of these keywords will be provided in the follow-up publication. Articles will be selected by scanning their titles and abstracts. Abstracts of the identified articles will be reviewed collectively by both the researchers. On reviewing abstracts potential studies will be identified. Full texts of the potential studies will be sourced and screened for the inclusion criteria. If the text of potential studies is not available, full texts will be requested from the authors. A third reviewer will be included to help with final selection of the studies. All disagreements within the team will be resolved by mutual discussions. Published as well as grey literature will be searched for potential articles. Reference lists of included articles will also be hand searched for potential studies. A PRISMA chart will be used to document the study selection procedure and a summary of the electronic search will be recorded using the format described in Table 2. We will use The Joanna Briggs Institute critical appraisal checklist for qualitative research to appraise the quality of final studies included in the scoping review.

Table 2 Summary of electronic search

Database	Keywords used	No of publications identified	No of publications included

For peer review only

Step 4: Data extraction

Data will be extracted from each included study using a systematic approach and measures will be taken to maintain uniformity in the data extraction process. A standardized data extraction form will be developed and used for charting the data, adopting the titles described by Arskey and O'Malley. [18] Data extraction will be limited to and focused on the research question. The variables to be included for data extraction are as indicated in Table 3.

Table 3: Data extraction form

Author and Date	
Title of the study	
Aim of the study	
Additional research questions/ Objectives	
Study design characteristics	
Participant characteristics	
Outcomes reported	
Most important finding:<br>- Positive comments<br>- Negative comments	
Other relevant findings :<br>- Positive comments<br>- Negative comments	
Conclusions	
Study limitations	
Authors recommendations	

Step 5: Reporting of the results

For peer review only

Synthesis of data is not typically a central objective for a scoping review. However, depending on the nature of the data collected we will provide a narrative synthesis or apply qualitative (thematic analysis) and quantitative methods (descriptive statistics such as percentage), to describe the extent and nature of the studies included. Charts and tables will also be used to map the study findings and provide an overview of the concepts, main sources and types of evidence in the research area under review.

Step 6: Consulting

Levac and colleagues [20] suggest that consultation be adopted to provide insight and input beyond the literature. To ensure a patient-centred approach, local patients receiving, involved in or affected by integrated care will be engaged throughout the process of the scoping review to improve the generalizability of the results and their responses and opinions will be incorporated into the study. To facilitate wider knowledge translation activities, the same patients and their families will be involved in subsequent sharing and discussion of the scoping review findings with other stakeholders.

Study Dissemination

The scoping review findings will be published in a peer-reviewed journal; presented at public forums and conferences; and will help determine the value of undertaking a full systematic review. The results from this scoping review will inform and guide the next phase of a multi-stage research study. The review findings will be made accessible to providers, policy makers and consumers to make effective use of the findings.

Discussion

The study aims at reviewing the literature on patients' perspectives, expectations and experiences of integrated care. This study aims at enhancing our understanding on how integrated care should look from a patient perspective. This study addresses an urgent need for establishing patient-centred care in the community setting in order to reduce the burden of fragmented care of patients, health systems and society. We intend to define "integrated care" from the patient perspective and to identify facilitators and barriers to patient-centred integrated care. It is anticipated that the findings from this review will enable health care providers, researchers and policy makers to more effectively tailor integrated care suited to patient needs.

For peer review only

Contributors: MMM conceived the idea, developed the research protocol and methods and drafted and edited the final manuscript. AM and GH aided in refining and developing the research question, study methods and made meaningful contributions to the drafting, editing of the manuscript. MMM, AM and GH all approved the final manuscript submitted.

Funding: The study is funded by The Hospital Research Foundation of South Australia.

Competing interests: The authors declare that they have no competing interests.

For peer review only

References:

1. Wang H, Dwyer-Lindgren L, Lofgren KT, *et al*. Age-specific and sex-specific mortality in 187 countries, 1970–2010: a systematic analysis for the Global Burden of Disease Study 2010. *Lancet*. 2013;380(9859):2071-94.

2. Oeppen J, Vaupel JW. Broken limits to life expectancy. *Science*. 2002;296(5570):1029-31.

3. Shaw S, Rosen R, Rumbold R. *What is integrated care? An overview of integrated care in the NHS*. London: Nuffield Trust, 2011.

4. World Health Organisation. *WHO Global Forum on Innovations for Ageing Populations Report of the forum*. 2013. Japan (accessed 15th July 2017). http://www.who.int/kobe_centre/publications/gfiap_report/en/

5. Calver J, Brameld KJ, Preen DB, *et al*. High-cost users of hospital beds in Western Australia: a population-based record linkage study. *Med J Aust*. 2006;184(8):393-7.

6. Downing A, Wilson R. Older people's use of accident and emergency services. *Age Ageing*.2005;34(1), 24–30.

7. Britt HC, Harrison CM, Miller GC, *et al*. Prevalence and patterns of multimorbidity in Australia. *Med J Aust*. 2008;189(2):72-7.

8. Di Pollina L, Guessous I, Petoud V, *et al*. Integrated care at home reduces unnecessary hospitalizations of community-dwelling frail older adults: a prospective controlled trial. *BMC Geriatr*. 2017;17(1):53.

9. Damery S, Flanagan S, Combes G. The effectiveness of interventions to achieve co-ordinated multidisciplinary care and reduce hospital use for people with chronic diseases: study protocol for a systematic review of reviews. *Syst Rev*. 2015;4(1):1-6.

10. Department of Health. *Supporting People with Long-term Conditions: an NHS and social care model to support local innovation and integration*. London: Department of Health, 2005.

11. Rollins, A.L., Wright-Berryman, J., Henry, N.H., *et al*. Managing physical and mental health conditions: Consumer perspectives on integrated care. *Soc. Work Mental Hlth*. 2017;*15*(1),66-79.

For peer review only

12. van Overveld, L.F.J., Takes, R.P., Braspenning, J.C., *et al.* Quality of Dutch integrated head and neck cancer care: *Meas. Eval.*2017;173-173.

13. Katz A, Mishler E. Close encounters: exemplars of process-oriented qualitative research in health care. *Qual. Res*. 2003; 3: 25–56.

14. World Health Organization, *WHO global strategy on people-centred and integrated health services: interim report.* 2015.

15. Berwick DM, Nolan TW, Whittington J. The triple aim: care, health, and cost. *Health Aff.* 2008;27(3):759-69.

16. Shaller D. Patient-Centered Care: *What Does it Take?* The Commonwealth Fund, 2007

17. Don Redding. The narrative for person-centred coordinated care, *J Integr Care.* 2013;21(6):315-325 https://doi.org/10.1108/JICA-06-2013-0018

18. Arksey H, O'Malley L. Scoping studies: towards a methodological framework. *Int J Soc Res Methodol*. 2005;8(1):19-32.

19. World Health Organization. Integrated health services–what and why. *Technical brief.* 2008; 1:1-8. (accessed 15th July 2017) http://www.who.int/servicedeliverysafety/areas/people-centred-care/en/

20. Levac, D, Colquhoun, H, O'Brien, KK. Scoping studies: advancing the methodology. *Implement Sci.* 2010 5:69 https://implementationscience.biomedcentral.com/articles/10.1186/1748-5908-5-69

For peer review only

BMJ Open

What integrated care means from an older person's perspective? A scoping review protocol

Journal:	*BMJ Open*
Manuscript ID	bmjopen-2017-019256.R1
Article Type:	Protocol
Date Submitted by the Author:	24-Nov-2017
Complete List of Authors:	Mittinty, Manasi; The University of Adelaide, Adelaide Nursing School<br>Marshall, Amy; The University of Adelaide, Adelaide Nursing School<br>Harvey, Gillian; University of Adelaide
**Primary Subject Heading**:	Geriatric medicine
Secondary Subject Heading:	Evidence based practice, Geriatric medicine, Health services research, Patient-centred medicine
Keywords:	integrated care, ageing population, older people, person-centred

SCHOLARONE™ Manuscripts

For peer review only

What integrated care means from an older person's perspective? A scoping review protocol

Manasi Murthy Mittinty[1], Amy Marshall[1] and Gillian Harvey[1].

[1] Adelaide Nursing School, The University of Adelaide, Adelaide, South Australia, 5000

Corresponding author: Manasi Murthy Mittinty
Email: manasi.mittinty@adelaide.edu.au
Address: Level 4, AHMS building, 57 North Terrace, Adelaide, South Australia, 5005

For peer review only

Abstract

Introduction: According to the 2013 WHO Global Forum on Innovation for Ageing Populations, disabilities and morbidities associated with ageing could be minimised by accessing preventive care. One way of improving management of multi-morbidity in the older population is through the provision of "integrated care". Although integrated care means different things to different people, it typically symbolises continuity in care, thus preventing older patients from falling through gaps in the health and care system. However, very little is known about patient expectations and experiences of integrated care, which is vital for developing and implementing better models of care. The proposed scoping review aims to map literature on older patients' views, expectations, experiences and perspectives of integrated care.

Methods and analysis: Multiple electronic databases including PubMed, Web of Science, Embase, PsychInfo, Google Scholar, Cochrane Library, CINHAL, and ProQuest Dissertations will be searched for appropriate articles between August–December 2017. Reference lists of selected articles will also be searched for similar articles. Two experienced researchers will conduct an initial search of the literature to identify relevant articles. Abstracts of the identified articles will be reviewed collectively by two researchers to identify potential further studies. Full texts of the potential studies will be sourced and screened for the inclusion criteria. Appropriate qualitative and quantitative methods will be used to extract data from each included study.

Ethics & Dissemination: The scoping review will synthesize findings from studies reporting on patients' views and expectations of integrated care. This review expects to find information relating to facilitators and barriers of integrated care from an older person's perspective. The findings from the review will be applied when working with stakeholders representing older people, health care, aged care and community providers, researchers and policy makers to develop and evaluate a more locally tailored and person-centred approach to integrated care.

Keywords: integrated care; ageing population; older people; person-centred

For peer review only

Strengths and limitations of the study

- This study will be the first scoping review to provide a comprehensive synthesis of what older people consider to be integrated care; what does it mean to them and what do they expect?
- This study will search all sources of literature covering peer-reviewed articles, unpublished reports, conference proceedings and bibliographies.
- Stakeholders involved in the provision of or affected by integrated care, including older people, health care providers, government organisations, carers and family members will be engaged throughout the review process.
- Scoping reviews are generally not considered to provide generalizable findings because of the lack of synthesis of results; conducting a thematic analysis of the available literature will go some way to providing further insight into the findings than descriptive data alone.
- As this review will incorporate studies from different health care settings, it may lack specificity.

For peer review only

For peer review only - http://bmjopen.bmj.com/site/about/guidelines.xhtml

Background:

In the last 40 years, life expectancy has increased significantly on a global level[1 2] with a resultant increase in the proportion of older people in the population. This shift in demographics has both benefits and challenges. Older people are a great social and cultural resource; they are also lower financial contributors and higher health care system users. As older people often suffer from multi-morbidity, they typically receive treatments from more than one health professional, consume multiple medications and have multiple organisations and service providers involved in their care.[3] As a result, older people tend to have more preventable acute hospitalisations,[4-6] which puts significant strain on the patients, their families, carers, and health care systems resulting in suboptimal health outcomes, reduced quality of life for patients and increased health care costs.[7] These acute hospitalizations are frequently linked with a lack of integrated care.[8] Integrated care is considered a logical way to organize health care to reduce demands on the local health services while generating a broad range of services for patients.

Providing integrated, person-centred care promises to improve patient experience while minimizing the unnecessary use of healthcare resources.[9] However, it represents a major challenge facing health service providers and policy makers.[10] Despite numerous attempts at integration, uncertainty remains about which approaches are most effective [9] or how to achieve an appropriate form of integration at a local level.

Though there is a growing body of scientific evidence relating to integrated care and its implications, very few studies [11 12] have summarized patients' perspectives and expectations of integrated care. There is a lack of comprehensive understanding of what older people themselves consider to be integrated care; what does it mean to them and what do they expect? Successful management of chronic conditions heavily depends upon matching clinical evidence with patient expectation and experience.[13] This reinforces the importance of understanding patients' perspectives and views of integrated care. The proposed scoping review aims to map literature on patients' views, expectations, experiences and perspectives of integrated care. This paper describes the protocol that will be followed for conducting the scoping review.

Study rationale:

Globally, a large number of heterogeneous, disease-specific and setting-specific models of integrated care are being implemented. However, it remains unclear the extent to which these

For peer review only - http://bmjopen.bmj.com/site/about/guidelines.xhtml

models have been validated, in terms of meeting a person-centred view of integrated care. The necessity to incorporate patients' views when building these models has been identified by several studies.[14-16] Currently, there is a paucity of information on what factors patients' perceive to support or hinder their care. To our knowledge, only one published study has synthesized patient experiences and expectations of integrated care.[17] In the absence of a clear person-centred definition of integrated care, the scoping review seeks to facilitate the development of such a definition. It will also provide insight into this poorly understood area of research, and help to map the characteristics of the primary research that currently exists, as well as identifying knowledge gaps.

The objective of the review is to systematically examine the range and scope of literature; and to determine enablers and barriers of integrated care from an older person's perspective. This work will constitute the first step in a multi-stage Knowledge Translation study, to be conducted in South Australia (SA). The scoping review will enable us to build an in-depth understanding of person- centred integrated care and the contextualised barriers and enablers. This information will frame the co-design, implementation and evaluation of a person-centred integrated model of care for older people who are frequent users of acute care. In turn this information will allow policy makers and healthcare providers to modify their services to patient demands and needs.

Research Methods:

Literature search

A pragmatic publication date cut-off point of June 2008 was adopted to focus on studies that were conducted after the publication of a working definition of integrated care by WHO.[14] The scoping review will use the six steps described by Arskey and O'Malley [18] for conducting a scoping review. These steps will be used as a guide to identify, select and review the literature. A PRISMA chart is attached as Appendix 1.

Step1: Identifying the research question

Integrated care is defined by the World Health Organization as, "*The management and delivery of health services so that clients receive a continuum of preventive and curative services, according to their needs over time and across different levels of the health system*". [19] This definition suggests that a wide array of health care activities – including, but not limited to, frequency of consultation, quality of consultation, location of medical services,

For peer review only - http://bmjopen.bmj.com/site/about/guidelines.xhtml

interaction with healthcare providers, satisfaction with the care provided to follow up plans - form integrated care. Therefore, an iterative approach will be adopted for refining the research questions. This approach will enable familiarization with the current literature on the topic; to synthesize knowledge and information from relevant studies while helping to identify the knowledge gaps. The broad research question that we will start with is:

"What are the findings of research on older patient views, perspectives, expectations and experiences of integrated care?"

Step 2: Identifying the relevant studies

The literature search will be performed from August 2017– December 2017, using PubMed, Web of Science, Embase, Google Scholar, Cochrane Library, CINHAL, and ProQuest Dissertations. Terms specific to each individual database will be used for searching articles. Selection criteria defined in Table 1 will be followed for inclusion or exclusion of articles in the scoping review.

Table 1: Selection criteria to be used for identifying studies.

Study selection criteria	• Articles in English language published between June 1, 2008-October 31st 2017<br>• Studies conducted or reporting only on human subjects<br>• Studies reporting on empirical, interpretive and critical research using any type of study methodology or study designs (case control study, observational study, surveys, research reports and case reports)<br>• Studies reporting on any types of health care setting including primary care, hospitals, allied health practices, or emergency departments
Participant selection criteria:	• Studies conducted or reporting only on participants aged 60 years and above<br>• There will be no limitation on upper age and gender of the participants<br>• There will be no limitations on geographical location of the study participants

For peer review only - http://bmjopen.bmj.com/site/about/guidelines.xhtml

Specific exclusion criteria:	• Studies reporting on non-human subjects<br>• Studies not reporting on individuals aged 60 years and above<br>• Studies reported in another language than English

Step 3: Study selection

Two experienced researchers will independently conduct an initial search of literature to identify relevant articles. A priori set of search terms will be formulated prior to conducting the initial search. A full list of these keywords will be provided in the follow-up publication. Articles will be selected by scanning their titles and abstracts. Abstracts of the identified articles will be reviewed collectively by both the researchers. On reviewing abstracts potential studies will be identified. Full texts of the potential studies will be sourced and screened for the inclusion criteria. If the text of potential studies is not available, full texts will be requested from the authors. A third reviewer will be included to help with final selection of the studies. All disagreements within the team will be resolved by mutual discussions. Published as well as grey literature will be searched for potential articles. Reference lists of included articles will also be hand searched for potential studies. A PRISMA chart will be used to document the study selection procedure and a summary of the electronic search will be recorded using the format described in Table 2. We will use The Joanna Briggs Institute critical appraisal checklist [20] (qualitative research) and the STROBE statement [21] (quantitative research) to appraise the quality of final studies included in the scoping review.

Table 2 Summary of electronic search

Database	Keywords used	No of publications identified	No of publications included

Step 4: Data extraction

For peer review only - http://bmjopen.bmj.com/site/about/guidelines.xhtml

Data will be extracted from each included study using a systematic approach and measures will be taken to maintain uniformity in the data extraction process. A standardized data extraction form will be developed and used for charting the data, adopting the titles described by Arskey and O'Malley. [18] Data extraction will be limited to and focused on the research question. The variables to be included for data extraction are as indicated in Table 3.

Table 3: Data extraction form

Author and Date	
Title of the study	
Aim of the study	
Additional research questions/ Objectives	
Study design characteristics	
Participant characteristics	
Outcomes reported	
Most important finding:	
Other relevant findings :	
Conclusions	
Study limitations	
Authors recommendations	

For peer review only

Step 5: Reporting of the results

Synthesis of data is not typically a central objective for a scoping review. However, depending on the nature of the data collected we will provide a narrative synthesis or apply qualitative (thematic analysis) and quantitative methods (descriptive statistics such as percentage), to describe the extent and nature of the studies included. Charts and tables will also be used to map the study findings and provide an overview of the concepts, main sources and types of evidence in the research area under review.

Step 6: Consulting

For peer review only - http://bmjopen.bmj.com/site/about/guidelines.xhtml

Levac and colleagues [22] suggest that consultation be adopted to provide insight and input beyond the literature. To ensure a person-centred approach, a local advocacy group working for older people will be engaged in the process of the scoping review. To facilitate wider knowledge translation activities, the scoping review findings will be disseminated among older patients, their families and other stakeholders.

Study Dissemination and Ethics

The scoping review findings will be published in a peer-reviewed journal; presented at public forums and conferences; and will help determine the value of undertaking a full systematic review. The results from this scoping review will inform and guide the next phase of a multi-stage knowledge translation study. The review findings will be made accessible to providers, policy makers and consumers to make effective use of the findings.

Discussion

The study aims to review the literature on patients' perspectives, expectations and experiences of integrated care. This study aims to enhance our understanding of integrated care from an older persons' perspective. As such, it addresses an urgent need for establishing person-centred care in the community setting in order to reduce the burden of fragmented care of patients, health systems and society. We intend to define "integrated care" from the patient perspective and to identify facilitators and barriers to person-centred integrated care. It is anticipated that the findings from this review will enable health care providers, researchers and policy makers to more effectively tailor integrated care suited to patient needs.

Contributors: MMM conceived the idea, developed the research protocol and methods and drafted and edited the final manuscript. AM and GH helped to refine and develop the research question, study methods and made meaningful contributions to the drafting, editing of the manuscript. MMM, AM and GH all approved the final manuscript submitted.

Funding: The study is funded by The Hospital Research Foundation of South Australia. The funding organisation has no role in the review process.

Competing interests: The authors declare that they have no competing interests.

References:

For peer review only - http://bmjopen.bmj.com/site/about/guidelines.xhtml

1. Wang H, Dwyer-Lindgren L, Lofgren KT, *et al*. Age-specific and sex-specific mortality in 187 countries, 1970–2010: a systematic analysis for the Global Burden of Disease Study 2010. *Lancet.* 2013;380(9859):2071-94.

2. Oeppen J, Vaupel JW. Broken limits to life expectancy. *Science.* 2002;296(5570):1029-31.

3. Shaw S, Rosen R, Rumbold R. *What is integrated care? An overview of integrated care in the NHS.* London: Nuffield Trust, 2011.

4. World Health Organisation. *WHO Global Forum on Innovations for Ageing Populations Report of the forum.* 2013. Japan (accessed 15th July 2017). http://www.who.int/kobe_centre/publications/gfiap_report/en/

5. Calver J, Brameld KJ, Preen DB, *et al.* High-cost users of hospital beds in Western Australia: a population-based record linkage study. *Med J Aust*. 2006;184(8):393-7.

6. Downing A, Wilson R. Older people's use of accident and emergency services. *Age Ageing*.2005;34(1), 24–30.

7. Britt HC, Harrison CM, Miller GC, *et al.* Prevalence and patterns of multimorbidity in Australia. *Med J Aust*. 2008;189(2):72-7.

8. Di Pollina L, Guessous I, Petoud V, *et al.* Integrated care at home reduces unnecessary hospitalizations of community-dwelling frail older adults: a prospective controlled trial. *BMC Geriatr*. 2017;17(1):53.

9. Damery S, Flanagan S, Combes G. The effectiveness of interventions to achieve co-ordinated multidisciplinary care and reduce hospital use for people with chronic diseases: study protocol for a systematic review of reviews. *Syst Rev.* 2015;4(1):1-6.

10. Department of Health. *Supporting People with Long-term Conditions: an NHS and social care model to support local innovation and integration.* London: Department of Health, 2005.

11. Rollins, A.L., Wright-Berryman, J., Henry, N.H., *et al.* Managing physical and mental health conditions: Consumer perspectives on integrated care. *Soc. Work Mental Hlth.* 2017;*15*(1),66-79.

For peer review only - http://bmjopen.bmj.com/site/about/guidelines.xhtml

12. van Overveld, L.F.J., Takes, R.P., Braspenning, J.C., *et al.* Quality of Dutch integrated head and neck cancer care: *Meas. Eval.*2017;173-173.

13. Katz A, Mishler E. Close encounters: exemplars of process-oriented qualitative research in health care. *Qual. Res*. 2003; 3: 25–56.

14. World Health Organization, *WHO global strategy on people-centred and integrated health services: interim report.* 2015.

15. Berwick DM, Nolan TW, Whittington J. The triple aim: care, health, and cost. *Health Aff.* 2008;27(3):759-69.

16. Shaller D. Patient-Centered Care: *What Does it Take?* The Commonwealth Fund, 2007

17. Don Redding. The narrative for person-centred coordinated care, *J Integr Care.* 2013;21(6):315-325 https://doi.org/10.1108/JICA-06-2013-0018

18. Arksey H, O'Malley L. Scoping studies: towards a methodological framework. *Int J Soc Res Methodol*. 2005;8(1):19-32.

19. World Health Organization. Integrated health services–what and why. *Technical brief.* 2008; 1:1-8. (accessed 15th July 2017) http://www.who.int/servicedeliverysafety/areas/people-centred-care/en/

20. Joanna Briggs Institute. *Critical Appraisal Checklist for Qualitative Research*. 2017: 1-6

21. von Elm E., Altman D.G., Egger M., et al. STROBE Initiative. The Strengthening the Reporting of Observational Studies in Epidemiology (STROBE) statement: guidelines for reporting observational studies. *J Clin Epidemiol*. 2008;61(4):344-9.

22. Levac, D, Colquhoun, H, O'Brien, KK. Scoping studies: advancing the methodology. *Implement Sci.* 2010 5:69 https://implementationscience.biomedcentral.com/articles/10.1186/1748-5908-5-69

For peer review only - http://bmjopen.bmj.com/site/about/guidelines.xhtml

Appendix 1- PRISMA-P Checklist

Section and topic	Page number
**Administrative Information**	
Title:	
Identification	1
Update	N/A
Registration	N/A
Authors:	
Contact	1
Contributions	9
Amendments	N/A
Support:	
Sources	9
Sponsor	9
Role of sponsor or funder	9
**Introduction**	
Rationale	4-5
Objectives	5
**Methods**	
Eligibility criteria	6-7
Information sources	6-7
Search Strategy	7-8
Study records:	
Data management	7-8
Selection process	7
Data collection process	8
Data items	
Outcomes and prioritization	8-9
Risk of bias in individual studies	
Data synthesis	8
Meta-bias(es)	N/A
Confidence in cumulative evidence	N/A

For peer review only

BMJ Open

What integrated care means from an older person's perspective? A scoping review protocol

Journal:	*BMJ Open*
Manuscript ID	bmjopen-2017-019256.R2
Article Type:	Protocol
Date Submitted by the Author:	21-Dec-2017
Complete List of Authors:	Mittinty, Manasi; The University of Adelaide, Adelaide Nursing School<br>Marshall, Amy; The University of Adelaide, Adelaide Nursing School<br>Harvey, Gillian; University of Adelaide
**Primary Subject Heading**:	Geriatric medicine
Secondary Subject Heading:	Evidence based practice, Geriatric medicine, Health services research, Patient-centred medicine
Keywords:	integrated care, ageing population, older people, person-centred

SCHOLARONE™
Manuscripts

For peer review only

What integrated care means from an older person's perspective? A scoping review protocol

Manasi Murthy Mittinty[1], Amy Marshall[1] and Gillian Harvey[1].

[1] Adelaide Nursing School, The University of Adelaide, Adelaide, South Australia, 5000

Corresponding author: Manasi Murthy Mittinty
Email: manasi.mittinty@adelaide.edu.au
Address: Level 4, AHMS building, 57 North Terrace, Adelaide, South Australia, 5005

For peer review only

For peer review only - http://bmjopen.bmj.com/site/about/guidelines.xhtml

Abstract

Introduction: According to the 2013 WHO Global Forum on Innovation for Ageing Populations, disabilities and morbidities associated with ageing could be minimised by accessing preventive care. One way of improving the management of multi-morbidity in the older population is through the provision of "integrated care". Although integrated care means different things to different people, it typically symbolises continuity in care, thus preventing older patients from falling through gaps in the health and care system. Many initiatives have attempted to improve the integration of care, however, these, are typically designed from a particular policy or system perspective. Relatively little is known about patient expectations and experiences of integrated care, which is vital for developing and implementing better models of care. The proposed scoping review aims to map literature on older patients' views, expectations, experiences and perspectives of integrated care.

Methods and analysis: Multiple electronic databases including PubMed, Web of Science, Embase, PsychInfo, Google Scholar, Cochrane Library, CINHAL, and ProQuest Dissertations will be searched for appropriate articles between August–December 2017. Reference lists of selected articles will also be searched for similar articles. Two experienced researchers will conduct an initial search of the literature to identify relevant articles. Abstracts of the identified articles will be reviewed collectively by two researchers to identify potential further studies. Full texts of the potential studies will be sourced and screened for the inclusion criteria. Appropriate qualitative and quantitative methods will be used to extract data from each included study.

Ethics & Dissemination: The scoping review will synthesize findings from studies reporting on patients' views and expectations of integrated care. This review expects to find information relating to facilitators and barriers of integrated care from an older person's perspective. The findings from the review will be applied when working with stakeholders representing older people, health care, aged care and community providers, researchers and policy makers to develop and evaluate a more locally tailored and person-centred approach to integrated care.

Keywords: integrated care; ageing population; older people; person-centred

For peer review only

For peer review only - http://bmjopen.bmj.com/site/about/guidelines.xhtml

Strengths and limitations of the study

- This study will be the first scoping review to provide a comprehensive synthesis of what older people consider to be integrated care; what does it mean to them and what do they expect?
- This study will search all sources of literature covering peer-reviewed articles, unpublished reports, conference proceedings and bibliographies.
- Stakeholders involved in the provision of or affected by integrated care, including older people, health care providers, government organisations, carers and family members will be engaged throughout the review process.
- Scoping reviews are generally not considered to provide generalizable findings because of the lack of synthesis of results; conducting a thematic analysis of the available literature will go some way to providing further insight into the findings than descriptive data alone.
- As this review will incorporate studies from different health care settings, it may lack specificity.

For peer review only

For peer review only - http://bmjopen.bmj.com/site/about/guidelines.xhtml

Background:

In the last 40 years, life expectancy has increased significantly on a global level[1 2] with a resultant increase in the proportion of older people in the population. This shift in demographics has both benefits and challenges. Older people are a great social and cultural resource; they are also lower financial contributors and higher health care system users. As older people often suffer from multi-morbidity, they typically receive treatments from more than one health professional, consume multiple medications and have multiple organisations and service providers involved in their care.[3] As a result, older people tend to experience more fragmented care, which can lead to more preventable acute hospitalisations,[4-6] placing additional strain on patients, their families, carers, and health care systems. In turn, this contributes to suboptimal health outcomes, reduced quality of life for patients and increased health care costs.[7 8]

Integrated care is proposed as a solution to both improve patient care and minimize the unnecessary use of healthcare resources.[9] However, it represents a major challenge facing health service providers and policy makers.[10] Despite numerous attempts at integration, uncertainty remains about which approaches are most effective[9] or how to achieve an appropriate form of integration at a local level. Empirical evidence highlights the complexities of achieving integrated care within pluralistic delivery systems with multiple stakeholders, varying cultures and different mechanisms of funding and governance. Whilst it is clear that there are no 'one-size-fits-all' solutions, successful approaches are typically bottom-up, driven by local need and with the support and engagement of all key stakeholders, including patients and their family. [11]

However, despite the growing body of scientific evidence relating to integrated care and its implications, few studies [12 13] have summarized patients' perspectives and expectations of integrated care. There is a lack of comprehensive understanding of what older people themselves consider to be integrated care; what does it mean to them and what do they expect? Some research suggests that there may be distinct differences between medical and patient narratives, with patients emphasising the importance of relational aspects of care and the everyday consequences of their condition, for example, in terms of functional impairment and feelings of vulnerability[14], important factors that influence a person's ability to manage and coordinate their own care. [13 15] This reinforces the importance of understanding patients' perspectives and views of integrated care. The proposed scoping review aims to map

For peer review only - http://bmjopen.bmj.com/site/about/guidelines.xhtml

literature on patients' views, expectations, experiences and perspectives of integrated care. This paper describes the protocol that will be followed for conducting the scoping review.

Study rationale:

Globally, a large number of heterogeneous, disease-specific and setting-specific models of integrated care are being implemented. However, it remains unclear the extent to which these models have been validated, in terms of meeting a person-centred view of integrated care. The necessity to incorporate patients' views when building these models has been identified by several studies.[16-18] Currently, there is a paucity of information on what factors patients' perceive to support or hinder their care. To our knowledge, only one published study has synthesized patient experiences and expectations of integrated care.[19] In the absence of a clear person-centred definition of integrated care, the scoping review seeks to facilitate the development of such a definition. It will also provide insight into this poorly understood area of research, and help to map the characteristics of the primary research that currently exists, as well as identifying knowledge gaps.

The objective of the scoping review is to systematically examine the range and scope of literature; and to determine enablers and barriers of integrated care from an older person's perspective. The review forms part of a larger program of work to co-produce and evaluate locally relevant approaches to improve integrated care for older people at risk of repeated hospitalisation. This program is informed by a conceptual framework known as the *co-knowledge translation framework* [20] (co-KT framework) (Figure 1). The framework was previously developed and tested in a population health study in a regional area of South Australia. [21] The basic tenet of the framework is that solutions to health service problems are best tackled by working with affected groups or communities to clarify and assess the problem and collectively develop, implement and evaluate appropriate evidence-informed solutions. Our pilot work looking at the coordination and continuity of care for older people in a defined geographical area of South Australia has highlighted opportunities for improving the integration of care between acute, primary and community care providers.[22] Combining this local knowledge with the co-KT framework, the scoping review forms part of our evidence gathering strategy to define the characteristics of integrated care from the perspective of those affected, that is, older people. The scoping review will be supplemented by a parallel study involving qualitative interviews with older people who have experienced frequent acute hospital presentations.

For peer review only - http://bmjopen.bmj.com/site/about/guidelines.xhtml

Figure 1: Co-KT Framework

Research Methods:

Literature search

A pragmatic publication date cut-off point of June 2008 was adopted to focus on studies that were conducted after the publication of a working definition of integrated care by WHO.[16] The scoping review will use the six steps described by Arskey and O'Malley [23] for conducting a scoping review. These steps will be used as a guide to identify, select and review the literature. A PRISMA chart is attached as Appendix 1.

Step1: Identifying the research question

Integrated care is defined by the World Health Organization as, "*The management and delivery of health services so that clients receive a continuum of preventive and curative services, according to their needs over time and across different levels of the health system*". [24] This definition suggests that a wide array of health care activities – including, but not limited to, frequency of consultation, quality of consultation, location of medical services, interaction with healthcare providers, satisfaction with the care provided to follow up plans - form integrated care. Therefore, an iterative approach will be adopted for refining the research questions. This approach will enable familiarization with the current literature on the topic; to synthesize knowledge and information from relevant studies while helping to identify the knowledge gaps. The broad research question that we will start with is:

"What are the findings of research on older patient views, perspectives, expectations and experiences of integrated care?"

Step 2: Identifying the relevant studies

The literature search will be performed from August 2017– December 2017, using PubMed, Web of Science, Embase, Google Scholar, Cochrane Library, CINHAL, and ProQuest Dissertations. Terms specific to each individual database will be used for searching articles. Selection criteria defined in Table 1 will be followed for inclusion or exclusion of articles in the scoping review.

Table 1: Selection criteria to be used for identifying studies.

For peer review only - http://bmjopen.bmj.com/site/about/guidelines.xhtml

Study selection criteria	• Articles in English language published between June 1, 2008-October 31st 2017<br>• Studies conducted or reporting only on human subjects<br>• Studies reporting on empirical, interpretive and critical research using any type of study methodology or study designs (case control study, observational study, surveys, research reports and case reports)<br>• Studies reporting on any types of health care setting including primary care, hospitals, allied health practices, or emergency departments
Participant selection criteria:	• Studies conducted or reporting only on participants aged 60 years and above<br>• There will be no limitation on upper age and gender of the participants<br>• There will be no limitations on geographical location of the study participants
Specific exclusion criteria:	• Studies reporting on non-human subjects<br>• Studies not reporting on individuals aged 60 years and above<br>• Studies reported in another language than English

Step 3: Study selection

Two experienced researchers will independently conduct an initial search of literature to identify relevant articles. A priori set of search terms will be formulated prior to conducting the initial search. A full list of these keywords will be provided in the follow-up publication. Articles will be selected by scanning their titles and abstracts. Abstracts of the identified articles will be reviewed collectively by both the researchers. On reviewing abstracts potential studies will be identified. Full texts of the potential studies will be sourced and screened for the inclusion criteria. If the text of potential studies is not available, full texts will be requested from the authors. A third reviewer will be included to help with final selection of the studies. All disagreements within the team will be resolved by mutual discussions. Published as well as grey literature will be searched for potential articles.

For peer review only - http://bmjopen.bmj.com/site/about/guidelines.xhtml

For peer review only

Reference lists of included articles will also be hand searched for potential studies. A PRISMA chart will be used to document the study selection procedure and a summary of the electronic search will be recorded using the format described in Table 2. We will use The Joanna Briggs Institute critical appraisal checklist [25] (qualitative research) and the STROBE statement [26] (quantitative research) to appraise the quality of final studies included in the scoping review.

Table 2 Summary of electronic search

Database	Keywords used	No of publications identified	No of publications included

Step 4: Data extraction

Data will be extracted from each included study using a systematic approach and measures will be taken to maintain uniformity in the data extraction process. A standardized data extraction form will be developed and used for charting the data, adopting the titles described by Arskey and O'Malley. [23] Data extraction will be limited to and focused on the research question. The variables to be included for data extraction are as indicated in Table 3.

Table 3: Data extraction form

Author and Date	
Title of the study	
Aim of the study	
Additional research questions/ Objectives	
Study design characteristics	
Participant characteristics	
Outcomes reported	
Most important finding:	

For peer review only - http://bmjopen.bmj.com/site/about/guidelines.xhtml

Other relevant findings :	
Conclusions	
Study limitations	
Authors recommendations	

Step 5: Reporting of the results

Synthesis of data is not typically a central objective for a scoping review. However, depending on the nature of the data collected we will provide a narrative synthesis or apply qualitative (thematic analysis) and quantitative methods (descriptive statistics such as percentage), to describe the extent and nature of the studies included. Charts and tables will also be used to map the study findings and provide an overview of the concepts, main sources and types of evidence in the research area under review.

Step 6: Consulting

Levac and colleagues [27] suggest that consultation be adopted to provide insight and input beyond the literature. To ensure a person-centred approach, a local advocacy group working for older people will be engaged in the process of the scoping review. To facilitate wider knowledge translation activities, the scoping review findings will be disseminated among older patients, their families and other stakeholders.

Study Dissemination and Ethics

The scoping review findings will be published in a peer-reviewed journal; presented at public forums and conferences; and will help determine the value of undertaking a full systematic review. The results from this scoping review will inform and guide the next phase of a multi-stage knowledge translation study. The review findings will be made accessible to providers, policy makers and consumers to make effective use of the findings.

Discussion

The study aims to review the literature on patients' perspectives, expectations and experiences of integrated care. This study aims to enhance our understanding of integrated care from an older persons' perspective. As such, it addresses an urgent need for establishing

For peer review only - http://bmjopen.bmj.com/site/about/guidelines.xhtml

person-centred care in the community setting in order to reduce the burden of fragmented care on patients, their informal and formal carers, health systems and society. We intend to define "integrated care" from the patient perspective and to identify facilitators and barriers to person-centred integrated care. It is anticipated that the findings from this review will enable health care providers, researchers and policy makers to more effectively tailor integrated care suited to patient needs.

Contributors: MMM conceived the idea, developed the research protocol and methods and drafted and edited the final manuscript. AM and GH helped to refine and develop the research question, study methods and made meaningful contributions to the drafting, editing of the manuscript. MMM, AM and GH all approved the final manuscript submitted.

Funding: The study is funded by The Hospital Research Foundation of South Australia. The funding organisation has no role in the review process.

Competing interests: The authors declare that they have no competing interests.

For peer review only

For peer review only - http://bmjopen.bmj.com/site/about/guidelines.xhtml

References:

1. Wang H, Dwyer-Lindgren L, Lofgren KT, *et al*. Age-specific and sex-specific mortality in 187 countries, 1970–2010: a systematic analysis for the Global Burden of Disease Study 2010. *Lancet* 2013;380:2071-94. doi: 10.1016/S0140-6736(12)61719-X
2. Oeppen J, Vaupel JW. Broken limits to life expectancy. *Science* 2002;296:1029-31. doi: 10.1126/science.1069675
3. Shaw S, Rosen R, Rumbold R. *What is integrated care? An overview of integrated care in the NHS.* London: Nuffield Trust, 2011. https://www.nuffieldtrust.org.uk/files/2017-01/what-is-integrated-care-report-web-final.pdf
4. World Health Organisation. *WHO Global Forum on Innovations for Ageing Populations Report of the forum.* 2013. Japan (accessed 15th July 2017). http://www.who.int/kobe_centre/publications/gfiap_report/en/
5. Calver J, Brameld KJ, Preen DB, *et al.* High-cost users of hospital beds in Western Australia: a population-based record linkage study. *Med J Aust* 2006;184:393-7. doi:
6. Downing A, Wilson R. Older people's use of accident and emergency services. *Age Ageing* 2005;34(1), 24–30. doi:10.1093/ageing/afh214
7. Britt HC, Harrison CM, Miller GC, *et al.* Prevalence and patterns of multimorbidity in Australia. *Med J Aust* 2008;189(2):72-7.
8. Di Pollina L, Guessous I, Petoud V, *et al.* Integrated care at home reduces unnecessary hospitalizations of community-dwelling frail older adults: a prospective controlled trial. *BMC Geriatr* 2017;17:53. doi: 10.1186/s12877-017-0449-9
9. Damery S, Flanagan S, Combes G. The effectiveness of interventions to achieve co-ordinated multidisciplinary care and reduce hospital use for people with chronic diseases: study protocol for a systematic review of reviews. *Syst Rev* 2015;4:1-6. doi: 10.1186/s13643-015-0055-x
10. Department of Health. *Supporting People with Long-term Conditions: an NHS and social care model to support local innovation and integration.* London: Department of Health, 2005. doi: 10.1186/s13643-015-0055-x
11. Wodchis WP, Dixon A, Anderson GM, et al. Integrating care for older people with complex needs: key insights and lessons from a seven-country cross-case analysis. *Int J Integr Care* 2015;15:e021. doi: 10.5334/ijic.2249

For peer review only - http://bmjopen.bmj.com/site/about/guidelines.xhtml

12. Rollins AL, Wright-Berryman J, Henry NH., *et al.* Managing physical and mental health conditions: Consumer perspectives on integrated care. *Soc. Work Mental Hlth* 2017;*15*,66-79. doi: 10.1080/15332985.2016.1173160

13. van Overveld LJ., Takes RP, Braspenning JC, *et al.* Quality of Dutch integrated head and neck cancer care: *Meas. Eval* 2017;173-173. doi: 10.1200/JCO.2017.35.8

14. Sheaff R, Halliday J, Byng R, et al. Bridging the discursive gap between lay and medical discourse in care coordination. *Sociol Health Illn* 2017;39:1019-34. doi: 10.1111/1467-9566.12553

15. Katz A, Mishler E. Close encounters: exemplars of process-oriented qualitative research in health care. *Qual. Res* 2003; 3: 25–56. doi: 10.1177/1468794103003001767

16. World Health Organization, *WHO global strategy on people-centred and integrated health services: interim report.* 2015. apps.who.int/iris/bitstream/10665/155002/1/WHO_HIS_SDS_2015.6_eng.pdf

17. Berwick DM, Nolan TW, Whittington J. The triple aim: care, health, and cost. *Health Aff* 2008;27:759-69. doi: 10.1377/hlthaff.273.759

18. Shaller D. Patient-Centered Care: *What Does it Take?* The Commonwealth Fund, 2007

19. Don Redding. The narrative for person-centred coordinated care, *J Integr Care* 2013;21:315-325. https://doi.org/10.1108/JICA-06-2013-0018

20. Powell K, Kitson A, Hoon E, et al. A study protocol for applying the co-creating knowledge translation framework to a population health study. Implement Sci. 2013;8:98. doi: 10.1186/1748-5908-8-98

21. Kitson, A, Powell, K, Hoon, E, et al. 'Knowledge translation within a population health study: how do you do it?', *Implement Sci* 2013; 8: 54. doi: 10.1186/1748-5908-8-54

22. Dollard J, Harvey G, Dent E, Hoon E, Karnon J, Kitson A, et al. Local solutions to improve health systems: towards an integrated care model for older people. Adelaide: School of Nursing, University of Adelaide, 2014.

23. Arksey H, O'Malley L. Scoping studies: towards a methodological framework. *Int J Soc Res Methodol* 2005;8:19-32. doi: 10.1080/1364557032000119616

24. World Health Organization. Integrated health services–what and why. *Technical brief* 2008; 1:1-8. (accessed 15th July 2017) http://www.who.int/servicedeliverysafety/areas/people-centred-care/en/

25. Joanna Briggs Institute. *Critical Appraisal Checklist for Qualitative Research*. 2017: 1-6

For peer review only - http://bmjopen.bmj.com/site/about/guidelines.xhtml

26. von Elm E., Altman D.G., Egger M., et al. STROBE Initiative. The Strengthening the Reporting of Observational Studies in Epidemiology (STROBE) statement: guidelines for reporting observational studies. *J Clin Epidemiol* 2008;61:344-9. doi: 10.1016/j.ijsu.2014.07.013

27. Levac, D, Colquhoun, H, O'Brien, KK. Scoping studies: advancing the methodology. *Implement Sci* 2010 5:69. doi: 10.1186/1748-5908-5-69

For peer review only

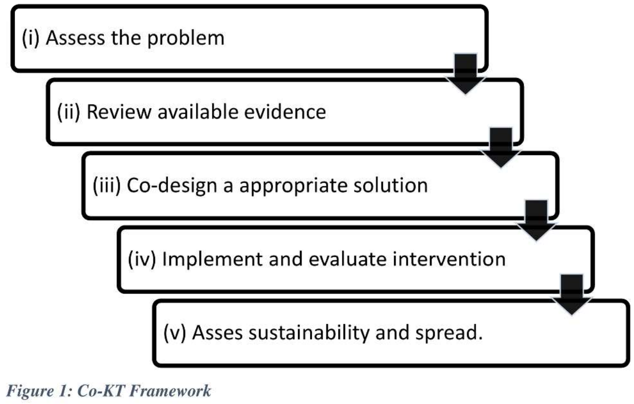

Figure 1: Co-KT Framework

210x297mm (300 x 300 DPI)

Appendix 1- PRISMA-P Checklist

Section and topic	Page number
**Administrative Information**	
Title:	
Identification	1
Update	N/A
Registration	N/A
Authors:	
Contact	1
Contributions	9
Amendments	N/A
Support:	
Sources	9
Sponsor	9
Role of sponsor or funder	9
**Introduction**	
Rationale	4-5
Objectives	5
**Methods**	
Eligibility criteria	6-7
Information sources	6-7
Search Strategy	7-8
Study records:	
Data management	7-8
Selection process	7
Data collection process	8
Data items	
Outcomes and prioritization	8-9
Risk of bias in individual studies	
Data synthesis	8
Meta-bias(es)	N/A
Confidence in cumulative evidence	N/A

For peer review only
